# The Long Noncoding RNA MALAT-1 Is Highly Expressed in Ovarian Cancer and Induces Cell Growth and Migration

**DOI:** 10.1371/journal.pone.0155250

**Published:** 2016-05-26

**Authors:** Yanqing Zhou, Xiaying Xu, Huabing Lv, Qirong Wen, Juan Li, Linyu Tan, Jianqi Li, Xiujie Sheng

**Affiliations:** Department of Obstetrics and Gynecology, The Third Affiliated Hospital and Key laboratory for Major Obstetric Diseases of Guangdong Province, Key Laboratory of Reproduction and Genetics of Guangdong Higher Education Institute in Guangdong Province, Guangzhou Medical University, Guangzhou, China; Peking University Cancer Hospital & Institute, CHINA

## Abstract

**Background:**

Metastasis associated in lung adenocarcinoma transcript-1 (MALAT-1) is overexpressed during cancer progression and promotes cell migration and invasion in many solid tumors. However, its role in ovarian cancer remains poorly understood.

**Methods:**

Expressions of MALAT-1 were detected in 37 normal ovarian tissues and 45 ovarian cancer tissues by reverse transcription polymerase chain reaction (RT-PCR). Cell proliferation was observed by CCK-8 assay; Flow cytometry was used to measure cell cycle and apoptosis; Cell migration was detected by transwell migration and invasion assay. In order to evaluate the function of MALAT-1, shRNA combined with DNA microarray and Functional enrichment analysis were performed to determine the transcriptional effects of MALAT-1 silencing in OVCAR3 cells. RNA and protein expression were measured by qRT-PCR and Western blotting, respectively.

**Results:**

We found that upregulation of MALAT-1 mRNA in ovarian cancer tissues and enhanced MALAT-1 expression was associated with FIGO stage. Knockdown of MALAT-1 expression in OVCAR3 cells inhibited cell proliferation, migration, and invasion, leading to G0/G1 cell cycle arrest and apoptosis. Overexpressed MALAT-1 expression in SKOV3 cells promoted cell proliferation, migration and invasion. Downregulation of MALAT-1 resulted in significant change of gene expression (at least 2-fold) in 449 genes, which regulate proliferation, cell cycle, and adhesion. As a consequence of MALAT-1 knockdown, MMP13 protein expression decreased, while the expression of MMP19 and ADAMTS1 was increased.

**Conclusions:**

The present study found that MALAT-1 is highly expressed in ovarian tumors. MALAT-1 promotes the growth and migration of ovarian cancer cells, suggesting that MALAT-1 may be an important contributor to ovarian cancer development.

## Introduction

Epithelial ovarian carcinoma (EOC) is the deadliest gynecologic malignancy, accounting for significant cancer death each year [1]. and [2]. Due to non-specific symptoms in the early stage of the disease and the lack of effective screening techniques in the clinic, the majority (up to 75%) of patients are diagnosed at advanced stages when the disease has already spread beyond the pelvis, resulting in a poor prognosis [3,4]. Despite continuous efforts to improve EOC diagnosis and treatment, tumor recurrence and metastasis remain major obstacles for successful treatment of EOC patients[5]. Thus, research on ovarian cancer early detection and improvement of current treatment strategies is urgently needed.

It is widely accepted that cancer development is a result of altered gene expression and/or structural changes of protein-coding genes. However, genome-wide sequencing data revealed that thousands of genes lack protein-coding capacity, while playing important roles in regulation of cell growth, survival and apoptosis, and tumorigenesis or other diseases [6]. Our research is focused on MALAT-1, originally identified in non-small cell lung cancer, is a large non-coding RNA that is closely associated with tumor metastasis and regulates the expression of various metastasis-associated genes[7]. MALAT-1 is located on chromosome 11q13.1, spans a 8.7kb genomic region, and is widely expressed in normal tissues, especially in the lung and pancreas [8],. MALAT-1 is specifically retained in the nuclear speckles [9] where pre-mRNA splicing factors are enriched and thus, may function as an assembly, storage, and/or modification compartment [10]. MALAT-1 has also been implicated in tumor progression [11]. Data has indicated that MALAT-1 was involved in cancer development through regulation of alternative splicing of its target genes [12] or gene expression [13,14]. MALAT-1 expression has been found to be up-regulated in many solid tumors, such as lung [8], liver [15], and prostate cancers [16] and has a tumor-promoting function. In this study, we measured MALAT-1 expression levels in primary normal ovary and ovarian tumor tissues and investigated the biological role of MALAT-1 in ovarian cancer pathogenesis.

## Materials and Methods

### 2.1 Tissue samples

37 epithelial specimens of normal ovary and 45 specimens of ovarian cancer tissues were obtained from The Third Affiliated Hospital of Guangzhou Medical University during 2009–2013. All ovarian cancer cases did not receive therapy prior to surgery and were diagnosed histologically according to the WHO book of gynecological pathology (Table 1). These tissue specimens were snap-frozen in liquid nitrogen and stored at -80°C until use. This study was approved by the institutional review of broad of Guangzhou Medical University and a written informed consent was obtained from all patients before participation of this study.

**Table 1:** Association of MALAT-1 expression with clinicopathological charateristics of ovarian cancer patients.

Variables	Number	MALAT-1 expression	p value
Age (years)			
<50	19	6.194	0.206
>50	26	2.925	0.206
Histological type			
Serous	21	5.537	0.666
Mucinous	24	3.227	0.666
Histological grade			
G1	17	2.24	0.354
G2/G3	28	4.031	0.354
FIGO stage			
I/II	21	1.009	0.000*
III/IV	24	7.189	0.000*
Ascites			
NO	21	1.069	0.175
YES	24	3.553	0.175

*p < 0.05.

### 2.2 Cell line and culture

Ovarian cancer cell line OVCAR3,SKOV3 were obtained from the American Type Culture Collection (ATCC, Manassas, VA, USA) and cultured in Dulbecco’s Modified Eagle’s Medium (DMEM) supplemented with 10% fetal bovine serum (FBS) and 1% streptomycin/penicillin at 37°C in 5% CO_2_. Cells were harvested in logarithmic phase of growth for all experiments described below.

### 2.3 RNA isolation and qRT-PCR

Total cellular RNA was isolated from tissues and cell line using Trizol reagent (Takara Bio, Inc., Shiga, Japan) and then reversely transcribed into cDNA using PrimeScript RT master Mix (Takara) according to the manufacturer’s instructions. qPCR was performed to determine the expression of MALAT-1, MMP-13, MMP-19, ADAMTS1, and β-actin mRNA using a SYBR GREEN MIX kit from Promega (Madison, WI, USA) according to the manufacturer’s protocols. Primers sequences are shown in Table 2. β-actin mRNA levels were used as an internal inference.

**Table 2:** Primer sequences used for qRT-PCR.

Gene	Forward primer	Reverse primer
MALAT-1	5'-AAAGCAAGGTCTCCCCACAAG-3'	5'-GGTCT GTGCAGATCAAAAGGCA-3'
MMP19	5'-CTGGATGCAGCTCTCTATTG-3'	5'-CTGCTGAAGTCAGTTCGG-3'
MMP13	5'-GGCAAGACTCTCCTGTTC-3'	5'-AGACAGCATCTACTTTATCACC-3'
NEXN	5'-TATATTGGGATTGGAGCGGG-3'	5'-CAACACTCAACACTGTATAGC-3'
ADAMTS1	5'-GGATCTTTGACCAGCACT-3'	5'-GCTGAGCCTTTCTCTCAT-3'
FRK	ACAATACCACTCCAGTAGCAGTGAA	GTGCAAACAGCATAAAGCTGGATAA
GBP2	AATAAGTACTACCAGGTGCCAAGGA	GCTTCTGCAGATTCAGCCTTTA
MAOB	AACTCTATGCCAAGGTTCTGGGTTC	CAGTCTCGGTGCCTGCAAAGTA
ANKRD1	AACCGCTATAAGATGATCCGACTCC	GAGTCTGTCGTTTGCCTCAGAATG
BHLHE41	TGAATGCATTGCTCAGCTGAAAG	TGATGCTGTTGCTCGGTTAAGG
MUC16	TCACCCAACTGGGCTTCTATGTC	GCAGGGTGATGTACTCTGAGGATG
TNFSF10	ATCGTGATCTTCACAGTGCTCCT	GGGGCTGTTCATACTCTCTTCGT
TSPAN2	AGTGTTAAGCTCCAGCTCATTGGAA	CAGCTCCTGTGACATTTGGTATGAA
FDCSP	GTTGGTTTCCCAGTCTCT	GGATATGGGTAAGGGAACAC
FXYD3	GCAAGATGTGGCCTGGAAG	GGTTCACCAGGCTGAGCAGA
MAP2K6	AGTGAAGATGTGCGATTTTGGA	TTGTCTGCTGGGAGTTGTGG
ACTB	5'-CCATCATGAAGTGTGACG-3'	5'-GCCGATCCACACGGAGTA-3'

### 2.4 Design and construction of MALAT-1vectors and gene transfection

To assess the role of MALAT-1 in ovarian cancer, we knocked down MALAT-1 expression using shRNAs and overexpressed MALAT-1 expression using pcDNA3.1(+)- MALAT-1 (a-MALAT-1). We designed and constructed four sets of MALAT-1 shRNA oligonucleotides (shM1-M4) and cloned them into the pGPU6/GFP/Neo vector (Jima, Shanghai, China). The sequences were MALAT-1-shM1, 5'-GCCGAAATAAATGAGAGAT GA-3'; shM2, 5'-GG CAGCTGTTAACAGATAAGT-3'; shM3, 5'-GCTGTGGAGTTCTTA AATATC-3'; shM4, 5'-GGGCTTCAGTGATGGGATAGT-3'. The pGPU6/GFP/Neo vector that contains a random DNA sequence was used as a scramble control (shNC). After cloning, amplification, and DNA sequencing, we transfected these vectors into OVCAR3 cells. In brief, cells were seeded in a 6-well plate and cultured overnight when reaching 70–80% confluency. OVCAR3 were transfected with shM1, shM2, shM3, shM4, or shNC using Lipofectamine 2000 (Invitrogen, Carlsbad, CA, USA) according to the manufacturer’s instructions. SKOV3 were transfected with pcDNA3.1 (+)- MALAT-1 and pcDNA3.1(SKOV3-NC). Cells were examined 48 h after transfection under an inverted fluorescence microscope to assess transfection efficiency. Cells were then harvested and subjected to qRT-PCR analysis of MALAT-1 expression. Because MALAT-1 shM3 was most effective in achieving knockdown of MALAT-1 expression, all subsequent experiments were performed using this shRNA.

### 2.5 Cell proliferation assay

To assess the effects of MALAT-1 in ovarian cancer cells, we first seeded cells into 96-well plates with 5 x 10^3^cells/well and cultured overnight. Cells were then transfected with MALAT-1-shM3, shNC, aMALAT-1 and SKOV3-NC. Cell proliferation was measured using the CCK-8 assay (Biyuntian, Shanghai, China) in 24h increments for up to 96 hours. In brief, 10 μl CCK-8 solution was added into each well of cell culture and the plates were further incubated for another 2.5 h. The optical density was then measured by FACS analysis at the absorbance of 450 nm. Experiments were performed in triplicate and repeated at least three times independently.

### 2.6 Transwell tumor cell migration and invasion assay

The Transwell chambers with 8 μm pores were obtained from Corning (Corning, NY, USA). Cells transfected were harvested, resuspended in DMEM without FBS at a concentration of 1 x 10^6^ cells in 100 μl, and then seeded into the upper chambers of the 24-well plate. The lower chambers were filled with 600 μl DMEM containing 10% FBS. Cells were then incubated for 24 h. At the end of the experiment, cells that migrated into the reverse side of the Transwell membrane were fixed with methanol, stained with crystal violet, and then counted under a light microscope at x100 magnification. An average of five visual fields was examined. For the tumor cell invasion assay, the Transwell membrane was pre-coated with 30 μl of Matrigel (1:3 mixed with PBS; BD Biosciences, Heidelberg, Germany) and proceeded the same as described above.

### 2.7 Cell cycle and apoptosis flow cytometry assay

Cells were harvested and resuspended at a density of 5–10 x 10^6^ cells/ml and then fixed with 75% ice-cold ethanol and stained with 400 μl propidium iodide (PI) solution (BestBio, Shanghai, China) for 30 min and then subjected to cycle analysis with a flow cytometer (BD). For apoptosis analysis, cells were stained with 5 μl of Annexin V-FITC (BD Biosciences) for 15 min and 5 μl PI for another 5 min before they were subjected to flow cytometry analysis.

### 2.8 Profiling of differential gene expression after MALAT-1 knockdown in ovarian cancer cells

Total RNA was extracted with the TRIZOL reagent and quantified by the NanoDrop ND-2000 (Thermo Scientific). The integrity of RNAs was assessed using Agilent Bioanalyzer 2100 (Agilent Technologies). cDNA libraries were constructed from samples with an RNA integrity number (RIN) ≥7.0. Total RNAs were transcribed to double strand cDNAs and then synthesized cRNAs. Next, 2nd cycle cDNAs were synthesized from cRNAs. Followed fragmentation and biotin labeling, the 2nd cycle cDNAs were hybridized onto the microarray. After washing and staining, the arrays were scanned by the Affymetrix Scanner 3000 (Affymetrix).

### 2.9 Gene expression analysis

Genespring software (version 12.5; Agilent Technologies) was used to complete the gene expression analysis. Differentially expressed genes (DEG) were identified using fold change as well as P value calculations. The threshold for selecting differentially expressed genes was a fold change> 2.0 and a P value < 0.05. KEGG and GO analysis were performed to determine the pathways and GO terms associated with differentially expressed mRNAs. Hierarchical Clustering (HCL) was performed to display the distinguishable gene' expression pattern among samples.

### 2.10 Analysis of mRNA expression of DEGs by qRT-PCR

To verify the microarray results, twelve differently expressed genes were chosen for qRT-PCR validation using a SYBR GREEN MIX kit(Promega, USA) and a FAST 7500 Real-Time PCR system following the manufacturer’s instructions, usingβ-actin as an internal control. Comparative quantification was determined by 2-ΔΔCt calculation method. Candidate genes andβ-actin gene were amplified in the same reaction and performed in triplicate. PCR primer sequences were listed in Table 2.

### 2.11 Protein extraction and Western blot

Total cellular protein was extracted from transfected cells and protein concentration was determined using a BCA protein assay kit (Beyotime, Beijing, China). These protein samples were resolved in 10% SDS denatured polyacrylamide gel and transferred onto PVDF membranes with a pore size of 0.45 μm (Millipore, Billerica, MA, USA). After blocking in 5% skim milk in Tris-based saline-Tween 20 (TBST) for 60 min at room temperature, membranes were incubated with mouse/rabbit anti-human antibodies at the recommended dilution [ADAMTS1 and MMP19 at a dilution of 1:1000, MMP13 at 1:500 (all from AbCam, Cambridge, MA, USA)] overnight at 4°C. After washed in TBST, the membranes were further incubated with a secondary anti-mouse (1:2500) or anti-rabbit (1:10000) antibody for 1 h. Enhanced chemiluminescence (ECL) solution was added onto the membranes and protein expression was quantified using The Laboratory Work Image Acquisition and Analysis Software (UVP, Upland, CA, USA). Glyceraldehyde 3-phosphate dehydrogenase (GAPDH) was used as a loading control.

### 2.12 Statistical analysis

All data were expressed as mean ± SD. Differences between two groups were assessed using the Fisher exact test or Student’s *t* test, while difference among multiple groups was analyzed using one–way ANOVA followed by Bonferroni’s multiple comparisons test. Differentially expressed genes after knockdown of MALAT-1 expression were assessed by using cDNA microarray (Affymetrix HTA 2.0), identified as fold changes and analyzed using Student’s *t* test. p<0.05 was considered statistically significant.

## Results

### 3.1 Differential expression of MALAT-1 mRNA in ovarian cancer tissue samples

**Fig 1:**
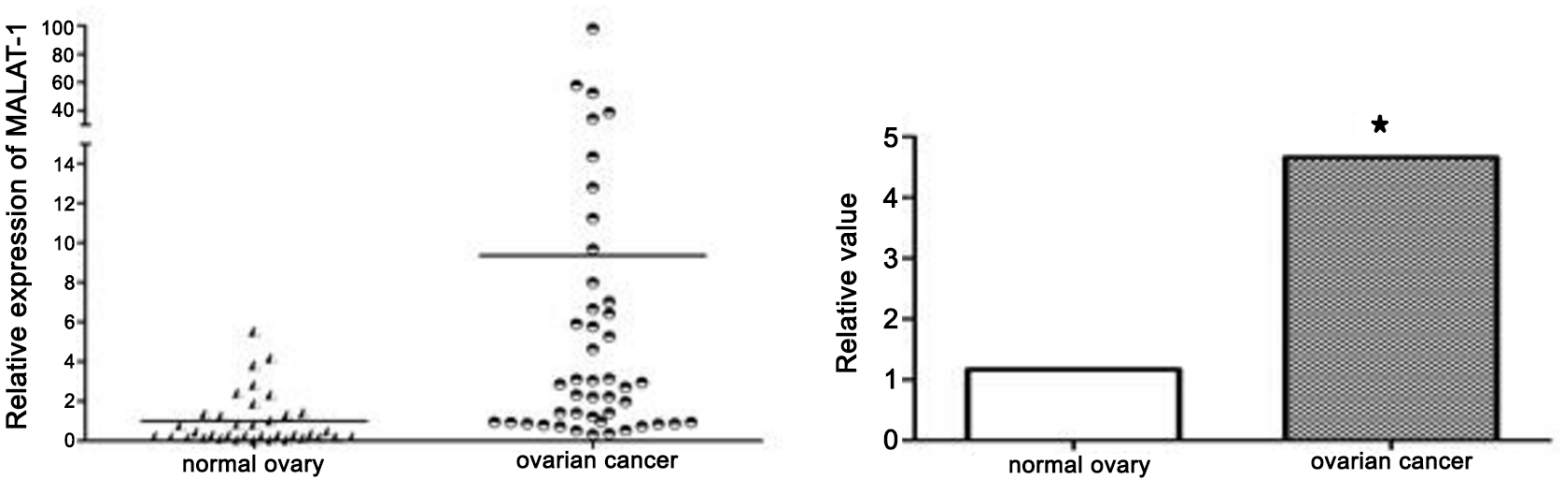
qRT-PCR analysis of MALAT-1 expression level in normal ovary and primary ovarian cancer tissues. MALAT-1 expression is significantly higher in ovarian cancer tissues. *p < 0.05.

MALAT-1 mRNA expression in control normal ovary and primary ovarian cancer tissues was evaluated by qRT-PCR and normalized to an internal control (β-actin). Expression of MALAT-1 in ovarian cancer tissues is significantly higher than that of in normal ovary (Fig 1). Furthermore, MALAT-1 expression is significantly associated with FIGO stages, but no association was identified between MALAT-1 expression and age, histological type, grade, or the presence of ascites (Table 1). These data suggested that high level of MALAT-1 expression was associated with ovarian cancer progression.

### 3.2 MALAT-1 are associated with the proliferation, migration, and invasion of ovarian cancer cells

**Fig 2:**
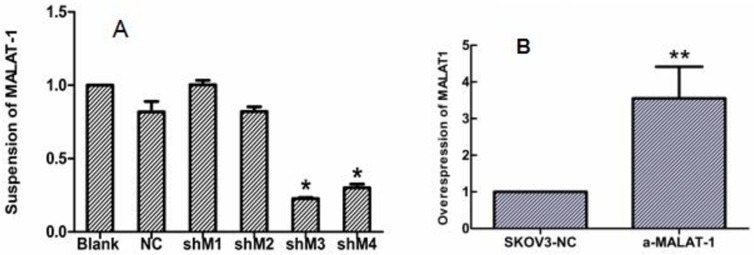
qRT-PCR analysis of MALAT-1 expression in transfected ovarian cells. **(A)** qRT-PCR analysis of MALAT-1 expression following shRNA knockdown. OVCAR3 cells were grown and transfected with four different MALAT-1 shRNA vectors (shM1 to shM4) and then subjected to qRT-PCR analysis. *p < 0.05 versus control groups.**(B)** SKOV3 cells were grown and transfected with pcDNA3.1 (+)- MALAT-1 and pcDNA3.1(NC) and subjected to qRT-PCR analysis, *p < 0.05 versus control groups.

**Fig 3:**
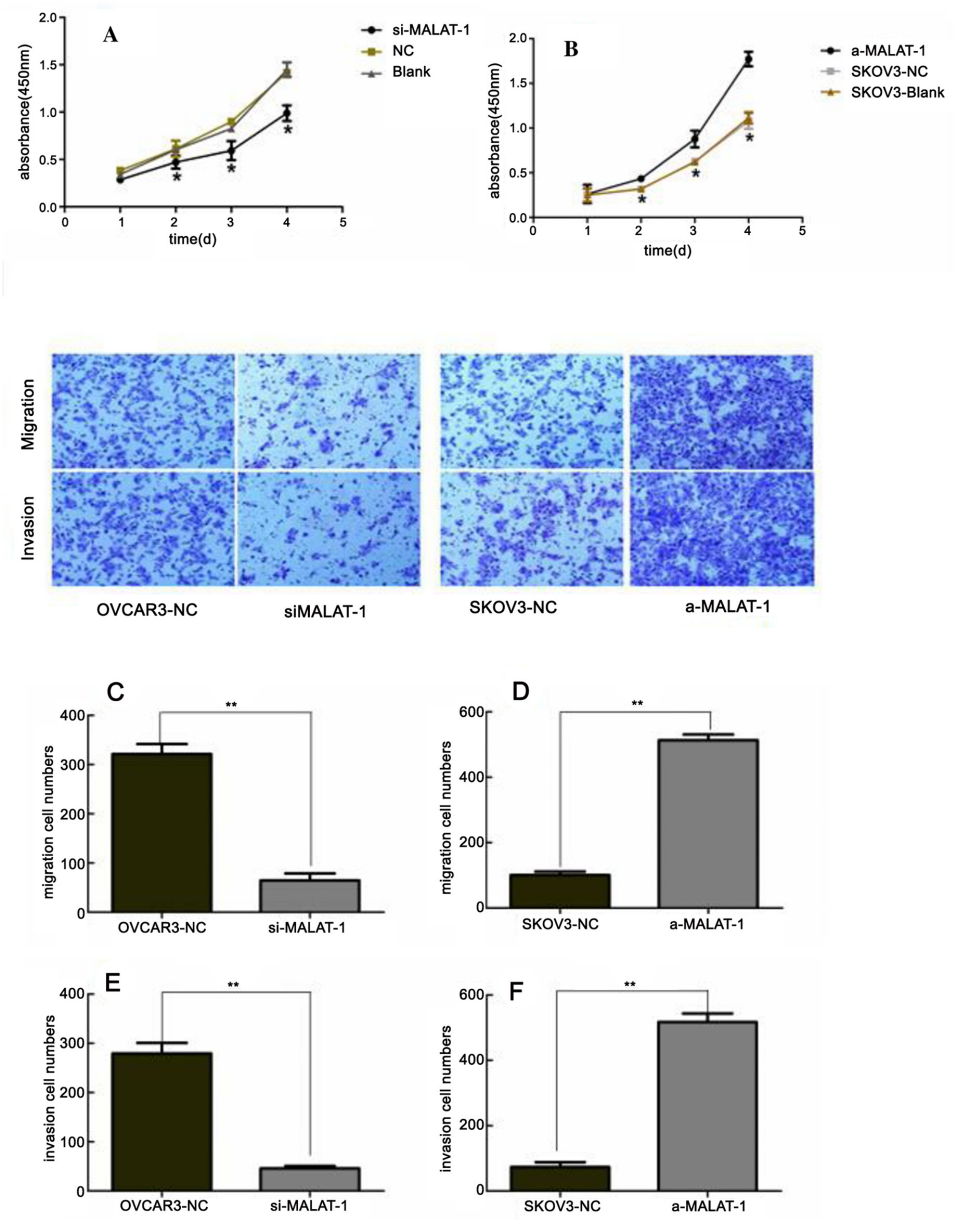
Suppression of MALAT-1 expression inhibits OVCAR3 cell growth, migration, and invasion, overexpression of MALAT-1 expression promotes SKOV3 cell growth, migration and invasion. **(A)** and **(B)** Cell proliferation CCK-8 assay. Ovarian cancer cells were grown and transfected and then subjected to CCK-8 assay. *p < 0.05 versus control groups. **(C)** and **(D)** Transwell tumor cell migration assay. Ovarian cancer cells were grown and transfected and then subjected to Transwell tumor cell migration assay.**(E)** and **(F)** Transwell tumor cell invasion assay. Ovarian cancer cells were grown and transfected and then subjected to Transwell tumor cell invasion assay **p < 0.0001 versus control groups.

Next, we constructed four shRNAs targeting MALAT-1. shRNA3 was the most effective in silencing MALAT-1 expression in OVCAR3 cells (Fig 2A). Therefore, we used this shRNA in all the following experiments. MALAT-1 knockdown significantly reduced ovarian cancer cell proliferation beginning at day 2 reaching the highest levels by day 5 (Fig 3A). In addition, MALAT-1 knockdown also significantly suppressed OVCAR3 migration and invasion capacity (Fig 3C and 3E). SKOV3 was highly expressed by transfected by pcDNA3.1 (+)- MALAT-1 and upregulation of MALAT-1 induced SKOV3 proliferation,migration and invasion (Fig 3B and 3D and 3F).

### 3.3 Knockdown of MALAT-1 expression induces apoptosis and arrests cell cycle at G0/G1 phase

**Fig 4:**
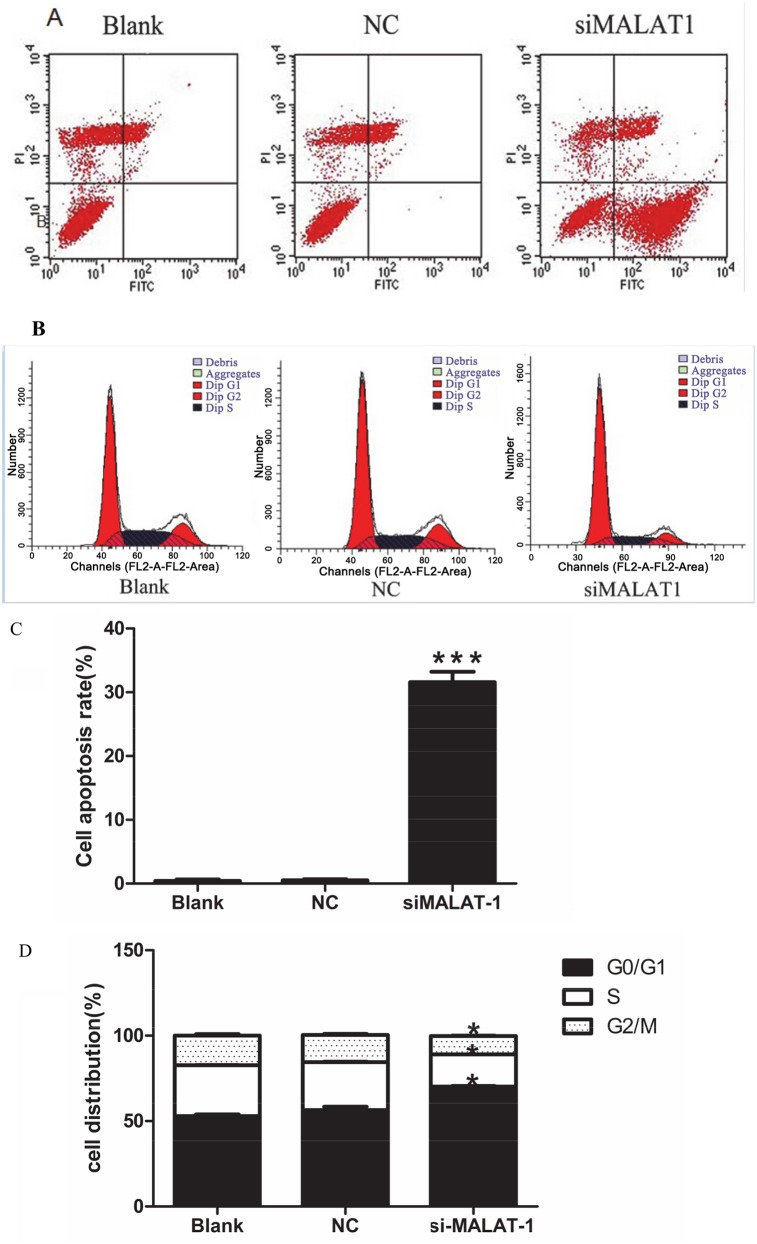
Effects of MALAT-1 knockdown on tumor cell apoptosis and cell cycle arrest. **(A)** Knockdown of MALAT-1 led to tumor cell apoptosis. OVCAR3 cells transfected with MALAT-1 shM3 were analyzed by flow cytometry for apoptosis. **(B)** Knockdown of MALAT-1 led to cell cycle arrest. OVCAR3 cells transfected with MALAT-1 shRNA3 were analyzed by flow cytometry analysis for cell cycle distribution. **(C)** Quantification of data in A. ***p < 0.0001 versus controls. **(D)** Quantification of data in B, *p < 0.05 versus controls.

Because knockdown of MALAT-1 inhibited cell proliferation (Fig 3A),we then examined MALAT-1 function in regulating ovarian cancer cell cycle progression and apoptosis. Our data demonstrated that the apoptotic cells in OVCAR3-shM3 cells were markedly increased to 17.8%, compared to 0.03% in the OVCAR3-shNC cells and 0.02% in parental OVCAR3 cells (p<0.001,Fig 4A and 4C). Furthermore, cell cycle analysis by flow cytometry demonstrated that knockdown of MALAT-1 expression arrested the OVCAR3-shM3 cells at the Go/G1 phase of the cell cycle and the percentages of cells in the S and G2/M phases were also decreased (P<0.05,Fig 4B and 4D). These data suggested that the inhibitory effect of MALAT-1 knockdown on OVCAR3 proliferation and growth might be caused by cell cycle arrest and increased apoptosis.

### 3.4 Downregulation of MALAT-1 impacts the expression of a large number of genes

**Fig 5:**
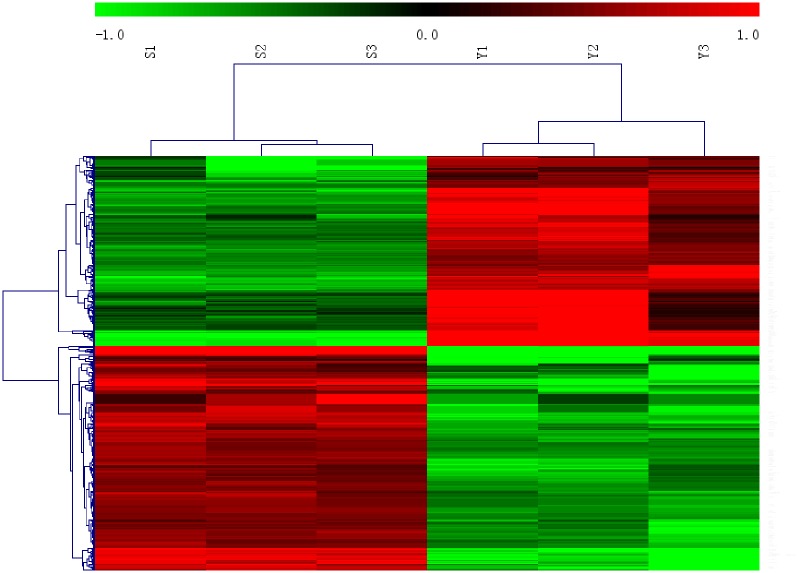
Identification of differently expressed genes between OVCAR3-NC and OVCAR3- shM3 cells. Unsupervised hierarchical clustering analysis of the global expression profiles of the differently expressed genes. Column represents sampl, and row represents gene, green represents a lower level gene expression and red represents a relative higher of gene expression.

**Fig 6:**
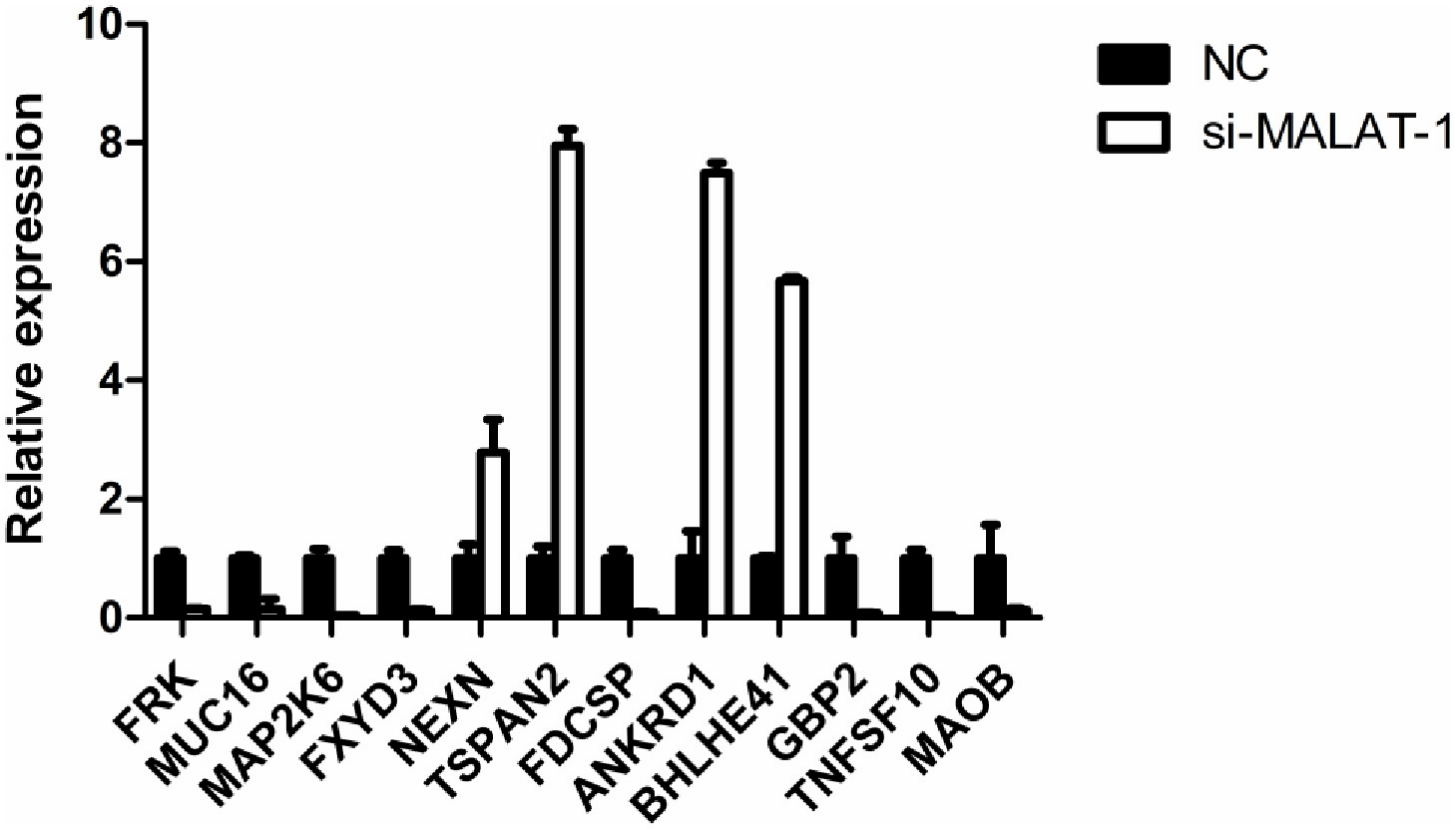
qRT-PCR confirmed the differently expressed genes identified by microarray from OVCAR-shNC and OVCAR3-shM3. The graph showed the average fold difference, FRK, MUC16,MAP2K6,FXYD3,FDCSP,MAOB,GBP2,TNFSF10 were down-regulated, NEXN, TSPAN2, NKRD1,BHLHE41 were up-regulated in OVCAR3-shM3 cells. (p<0.05).

The gene expression profiles of OVCAR3-shNC and OVCAR3-shM3 cells were analyzed by microarray analysis, and 449 significantly differentially expressed genes (>2-fold) were identified. Among them, 206 genes were down-regulated, while 243 genes were up-regulated in ovarian cancer cells with shRNA-mediated MALAT -1-silencing compared to the control OVCAR3 cells. A distinct list of genes are regulated by MALAT-1 as demonstrated by unsupervised hierarchical clustering (Fig 5A). We then performed qRT-PCR for 12 randomly selected genesto confirm the microarray results. The qRT-PCR results are highly comparable to the results from cDNA microarray analysis, in which *FRK*, *MUC16*, *MAP2K6*, *FXYD3*, *FDCSP*, *MAOB*, *GBP2*, and *TNFSF10* were down- regulated, *NEXN*, *TSPAN2*, *ANKRD1*, and *BHLHE41* were up-regulated in OVCAR3-shM3 cells compared to the shNC cells (Fig 6). Furthermore, GO and KEGG enrichment analysis of the most up- and down-regulated genes respectively, identified significantly over-represented functions highlighting a role of MALAT -1 relating to invasion and metastasis of ovarian cancer cells. Furthermore regul ation of transcription, positive regulation of cell proliferation,cell cycle,cell growth and cell adhesion by MAPK signaling or P53 signaling pathways were affected by MALAT-1 expression.(S1–S4 Figs).

### 3.5 MALAT-1 may involved in ovarian cancer by regulating the expression of *MMP13*, *MMP19*, *ADAMTS1*

**Fig 7:**
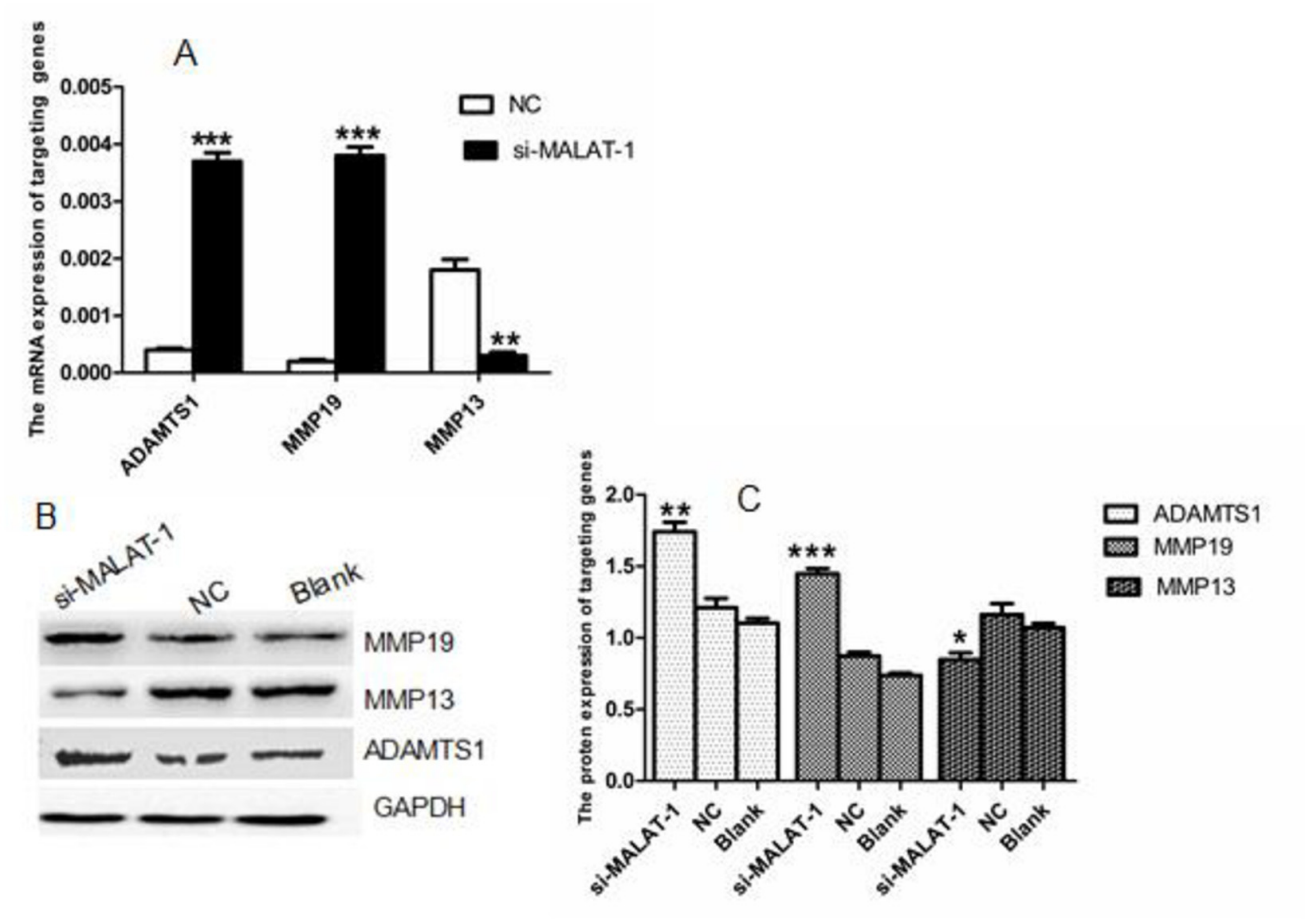
Extracellular matrix proteins are regulated by MALAT-1. **(A)** mRNA expression of ADAMTS1, MMP13, and MMP19 are regulated by MALAT-1. *p<0.05 versus control groups. **(B)**ADAMTS1, MMP13, and MMP19 protein expression are altered as a result of MALAT-1 knockdown. **(C)** Quantification of B. *p < 0.05 versus the control.

Among these DEGs, 79 genes were significantly up-regulated or down-regulated by at least of 3-fold. According to the past studies, *MMP19*, *ADAMTS1*and *MMP13* genes are closely associated with the metastasis and development of malignant tumors. We then performed qRT-PCR and Western blotting to confirm the expression of *MMP19*, *ADAMTS1* and *MMP13* genes between OVCAR3-shM3 and OVCAR3-shNC cells. The mRNA expression of *MMP13* is downregulated in the shM3 cells,while the expression of *MMP19 and ADAMTS1* was increased (**P<0.01, Fig 7A). Protein expression was consistent with mRNA results (Fig 7B). Suppression of MALAT-1 resulted in downregulating the expression of *MMP13* and up-regulating the expression of *MMP19 and ADAMTS1* genes, indicating that MALAT-1 may involved in ovarian cancer by regulating the expression of the *MMP13*, *MMP19*, *and ADAMTS1* genes.

## Discussion

In the human genome, DNA sequencing data has demonstrated that although thousands of genes lack protein-coding capacity, they could nonetheless play important roles in regulating cell growth, survival, apoptosis, and tumorigenesis [6]. Non-coding RNA can be classified according to their size into miRNAs (22 nt), piRNAs (18–30 nt), short translational-regulatory RNAs (100–200nt), and much longer ncRNAs (up to 10,000 nt)[17]. The long non-coding RNA with a length of >200 nt gained substantial attention recently. In the current study, we examined MALAT-1 expression in epithelial ovarian cancer tissues and investigated the role of MALAT-1 in the regulation of tumor cell migration and invasion in vitro. We found that MALAT-1 mRNA was overexpressed in tumor tissuesand MALAT-1 expression was associated with ovarian cancer progression. Knockdown of MALAT-1 expression suppressed tumor cell proliferation, migration, and invasion, arrested cells at the G0/G1 phase of cell cycle, and induced apoptosis. We further identified 79 genes whose expression was significantly altered (> 3-fold) following MALAT-1 knockdown. Interestingly, three of these genes, *MMP19*, *ADAMTS1*, and *MMP13*, have been previously implicated in the regulation of angiogenesis, extra-cellular matrix, cell adhesion, and cancer progression. Thus, our data suggests that overexpression of MALAT-1 might promote ovarian cancer progression by regulating the expression of *MMP19*, *ADAMTS1* and *MMP13* genes.

Metastasis is the main cause of mortality in cancer patients. The underlying mechanism for tumor metastasis and recurrence is very complex, including tumor cell adhesion, invasion, intravasation, circulation, extravasation, and growth in distant organizations [18]. Changes in intracellular signaling pathways that control cell proliferation and apoptosis as well as extracellular secretion of proteins that are involved in cell adhesion, migration, and proteolysis are of paramount importance to cancer metastasis [19]. Recently, the function of non-coding RNA, such as MALAT-1, during metastasis is starting to be appreciated [8, 14]. In gynecological malignancies, ovarian cancer is usually diagnosed as a metastatic disease. Our current study demonstrates that upregulation of MALAT-1 is associated with ovarian cancer progression. Indeed, previous studies have shown that MALAT-1 was significantly up-regulated in several types of cancers, including lung, liver, pancreatic, and prostate cancers. Moreover, MALAT-1 has served as a metastatic and recurrence biomarker for non-small cell lung cancer and hepatocellular cancer [15]. However, future studies with larger sample size are required to confirm our results.

Previous studies showed that tumor cells were capable of producing factors that induce tumor angiogenesis, as well as metalloproteinases and related molecules to degrade extracellular matrix [20]. Our current study showed MALAT-1 knockdown modulated the expression of MMP13 and MMP19, members of the matrix metalloproteinase (MMP) family of proteins that are involved in altered extracellular matrix metabolism, tumor progression and metastasis [21, 22]. Some previous studies showed that MMP13 is abnormally expressed in some solid tumors, such as papillary thyroid carcinoma and colorectal cancer and is associated with progression and metastasis [23,24]. In contrast, MMP19, which displays unique structural characteristics, plays a dual role in tissues. On the one hand, MMP19 deficiency increased the early angiogenic response, acting as a negative regulator of invasion. However, MMP-19, which is up-regulated in various cancer tissues, facilitates tumor invasion [25]. Consistent with the anti-angiogenic efficacy of MMP19, several reports have demonstrated that metallopeptidase with thrombospondin type-1 motif (ADAMTS1), which was up-regulated in MALAT-1-silencing cells to inhibit angiogenesis [26–28]^,^ and decreased ADAMTS1 stimulated migration and invasion of breast cancer cells [29].

To date, there is no report implicating MALAT-1 in the regulation of MMPs or ADAMTS1 expression in ovarian cancer. Nonetheless, based on our current data, we hypothesize that the efficacy of MALAT-1 in promoting ovarian cancer progression could be mediated by up-regulation of MMP13 and down-regulation of MMP19 and ADAMTS1.

To further elucidate the molecular mechanisms of MALAT-1-induced metastasis, we identified genes regulated by MALAT-1 expression, by comparing shRNA-inhibited versus control ovarian cancer cells. Further exploration of the target genes and signaling pathway will provide new insight into the molecular mechanisms of MALAT-1 involved in ovarian cancer progression and will aid in construction of a stronger prediction and prevention rule in ovarian cancer.

## Supporting Information

S1 Fig GO enrichment analysis of biological process of up-regulated and down-regulated genes. (JPG)

S2 Fig GO enrichment analysis of cellular component of up-regulated and down-regulated transcripts. (JPG)

S3 Fig GO enrichment analysis of molecular functions of up-regulated and down-regulated transcripts. (JPG)

S4 Fig KEGG enrichment analysis (pathway analysis). (JPG)
