# Supplementary figures and images for: The Long Noncoding RNA MALAT-1 Is Highly Expressed in Ovarian Cancer and Induces Cell Growth and Migration
Source: PLoS One. 2016 May 26;11(5):e0155250. doi: 10.1371/journal.pone.0155250 (PMC4881927; doi:10.1371/journal.pone.0155250)

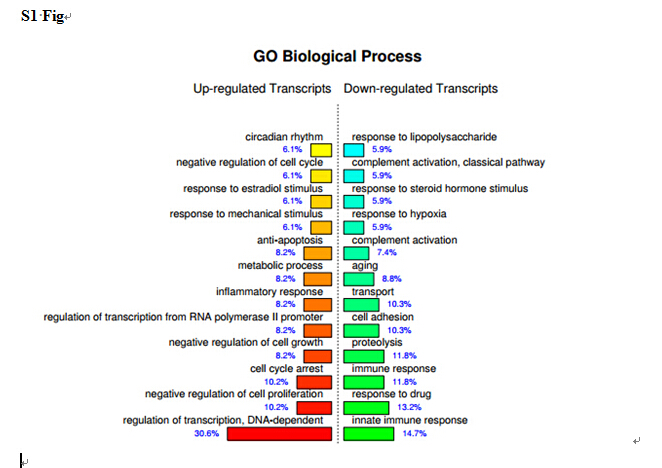

Supplement: S1 Fig — (JPG) [file pone.0155250.s001.jpg]

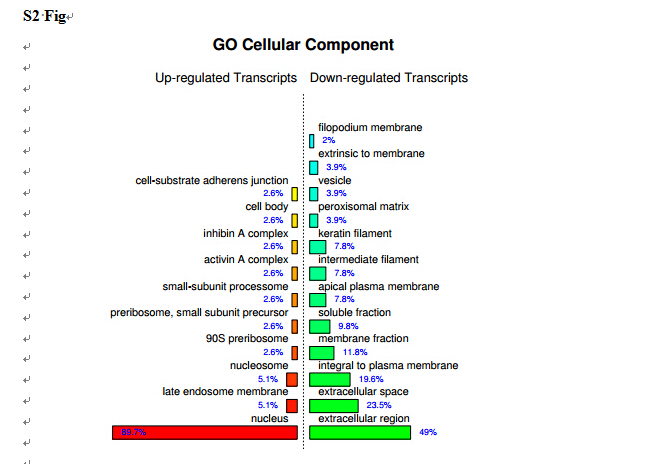

Supplement: S2 Fig — (JPG) [file pone.0155250.s002.jpg]

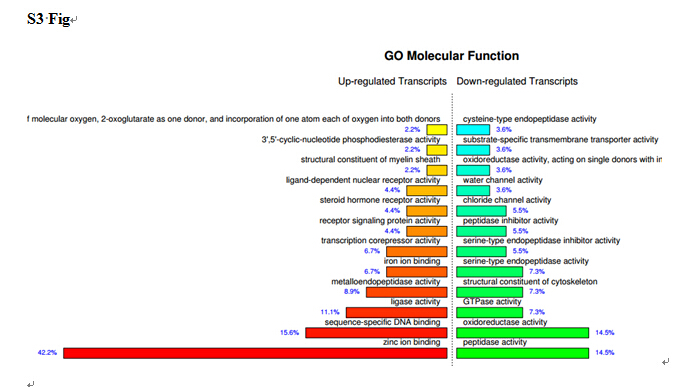

Supplement: S3 Fig — (JPG) [file pone.0155250.s003.jpg]

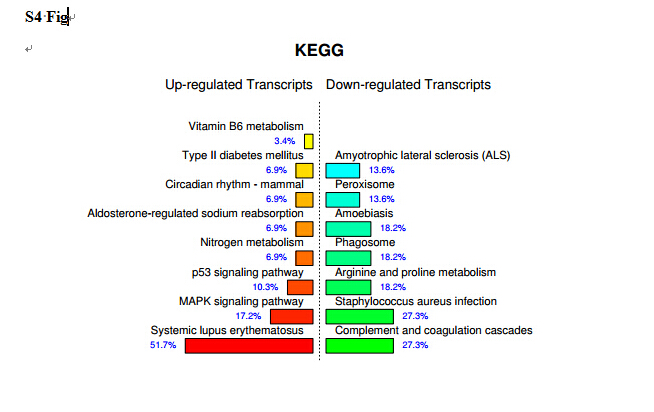

Supplement: S4 Fig — (JPG) [file pone.0155250.s004.jpg]
